# Metabolic Syndrome in Middle Eastern Patients with Atherosclerotic Cardiovascular Disease: A High Burden Driven by Cumulative Risk Factors

**DOI:** 10.3390/jcdd13060240

**Published:** 2026-05-31

**Authors:** Osama Alkouri, Walid Al-Qerem, Mohamad Jarrah, Ghaleb Alharbi, Nour Ali Alrida, Rahma Musaed Alabkal, Ayman Jaber Hammoudeh, Mohamed Ezzelregal Abdelgawad, Abdulkareem Alshehri, Abdullah Yaqoub Hasan, Mohannad AbuRuz, Fatma Refaat Ahmed, Mohammed Aldalaykeh

**Affiliations:** 1 Faculty of Nursing, Yarmouk University, P.O. Box 566, Irbid 21163, Jordan; o.alkouri@yu.edu.jo (O.A.); nour.alrida@yu.edu.jo (N.A.A.); mohannadeid@yahoo.com (M.A.); 2 Faculty of Pharmacy, Al-Zaytoonah University of Jordan, Amman 11733, Jordan; waleed.qirim@zuj.edu.jo; 3 Department of Internal Medicine, Faculty of Medicine, Jordan University of Science and Technology, Irbid 21110, Jordan; mijarrah@just.edu.jo; 4 Department of Clinical Pharmacy, College of Pharmacy, Shaqra University, Shaqra 11961, Saudi Arabia; g.alharbi@su.edu.sa; 5 Department of Pharmacy, Ministry of Health, Kuwait City 13001, Kuwait; ralabkal@hotmail.com; 6 Department of Cardiology, Istishari Hospital, Amman 11184, Jordan; hammoudeh_ayman@yahoo.com; 7 Medical Surgical Nursing Department, College of Nursing, Jouf University, Sakaka 72388, Saudi Arabia; meabedlgawad@ju.edu.sa; 8 Advanced Diagnostic and Therapeutic Institute, King Abdulaziz City for Science and Technology (KACST), Riyadh 12354, Saudi Arabia; abalshehri@kacst.gov.sa; 9 College of Nursing, The Public Authority for Applied Education and Training, Kuwait City 72853, Kuwait; ay.hasan@paaet.edu.kw; 10 College of Health Sciences, University of Sharjah, Sharjah 27272, United Arab Emirates; fahmed@sharjah.ac.ae; 11 College of Nursing, QU Health Sector, Qatar University, Doha P.O. Box 2713, Qatar

**Keywords:** SMuRF-less, multimorbidity, lipid abnormalities, type 2 diabetes, hypertension, Middle East epidemiology

## Abstract

Background: Metabolic syndrome (MS), characterized by a constellation of interrelated cardiometabolic abnormalities, markedly amplifies cardiovascular risk. Despite the high prevalence of atherosclerotic cardiovascular disease (ASCVD) in the Middle East, evidence regarding the burden and determinants of MS in this high-risk population remains limited. This study aimed to estimate the prevalence of MS and identify its independent predictors among Middle Eastern patients with established ASCVD. Methods: This comprehensive analysis integrated data from two complementary sources: a prospective cohort derived from the Jordan SMuRF-less Study, which enrolled adults (≥18 years) with confirmed ASCVD across nine centers in Jordan, and a pooled retrospective dataset from six regional cardiovascular registries. Standardized case report forms were used to collect demographic, clinical, and laboratory data. Participants were stratified according to the number of standard modifiable risk factors (SMuRFs) into three categories (0, 1–2, and 3–4 SMuRFs). Multivariable logistic regression analysis was conducted to determine independent predictors of MS. Results: Among 1016 patients with ASCVD, MS was present in 42.7% of the cohort. The prevalence of MS demonstrated a significant graded increase with higher SMuRF burden, rising from 2.2% in patients without SMuRFs to 28.3% in those with one to two SMuRFs and 62.2% in those with three to four SMuRFs (*p* < 0.001). Patients with MS were significantly older and exhibited higher body mass index and triglyceride levels, lower high-density lipoprotein cholesterol, and a greater prevalence of hypertension, diabetes mellitus, dyslipidemia, chronic kidney disease, and heart failure (all *p* < 0.001). Independent predictors of MS included advanced age, diabetes mellitus, hypertension, chronic kidney disease, heart failure, elevated body mass index, and increased triglyceride levels. In contrast, higher HDL cholesterol and smoking were inversely associated with MS. Conclusions: MS is highly prevalent among Middle Eastern patients with ASCVD and is strongly associated with cumulative SMuRF burden in a graded manner. These findings highlight the urgent need for targeted, region-specific strategies focusing on early identification and comprehensive management of cardiometabolic risk in this vulnerable population.

## 1. Introduction

Cardiovascular diseases (CVDs) continue to be the most significant cause of death and disability globally, imposing a considerable burden on individuals, healthcare systems, and economies [1]. Among this wide range of conditions, ASCVDs, encompassing myocardial infarction, stroke, and peripheral arterial disease, are responsible for the majority of cases [2,3]. Although considerable progress has been made in prevention and treatment approaches, major disparities exist, especially in populations with a high prevalence of metabolic risk factors [4,5].

Metabolic syndrome, a collection of conditions that include hypertension, dyslipidemia, insulin resistance, and central obesity, has been identified as a powerful predictor of cardiovascular events and poor health outcomes [6]. The relationship between MS and ASCVD is well established, such that MS not only elevates the risk for first cardiovascular events but also worsens the course of the disease and complicates recovery [7,8]. Additionally, a family history of premature cardiovascular disease also increases risk, suggesting a complex interaction between genetic, metabolic, and behavioral determinants [9].

In recent years, SMuRFs (standard modifiable risk factors), including smoking, diabetes mellitus, hypertension, and dyslipidemia, have become increasingly acknowledged as a practical approach to understanding and categorizing cardiovascular risk [10,11]. Although individual elements of SMuRFs are well known, there is limited knowledge of the association between the overall burden of each component and the presence of MS, especially in Middle Eastern populations, where lifestyle change and urbanization have contributed to rising trends of obesity, diabetes, and cardiovascular disease [12,13].

Despite the urgent necessity, investigations into the relationship between SMuRF collection, MS, and ASCVD outcomes among Middle Eastern populations remain scarce. Understanding this relationship is significant for developing targeted prevention and intervention programs adapted to these groups’ individual risk profiles. Therefore, the current study was designed to evaluate the prevalence of MS based on the burden of SMuRFs in Middle Eastern patients with ASCVD and a family history of premature CVD. The study also aimed to investigate demographic, clinical, metabolic, and pharmacological characteristics of MS and independent predictors of MS in this high-risk group. Through these findings, this study aims to contribute to a better understanding of cardiovascular risk stratification and prevention in Middle Eastern populations.

## 2. Materials and Methods

### 2.1. Study Design

This study incorporated data from two principal sources. The primary dataset originated from a prospective cohort of patients aged 18 years or older, diagnosed with ASCVD, and enrolled in the Jordan SMuRF-less Study (ClinicalTrials.gov Identifier: NCT06199869) between 10 January and 20 August 2024. Participants were recruited from nine healthcare facilities distributed across Jordan, including a private academic hospital, three Ministry of Health institutions, three community-based hospitals, and two university-affiliated hospitals.

The second dataset was established through a retrospective, post hoc analysis of ASCVD cases drawn from six major cardiovascular registries previously conducted within the Middle East region [11,14,15,16,17,18,19]. These included the First Jordan Percutaneous Coronary Intervention Registry (NCT01841346) (2426 patients) [14], the Surviving a Decade or More after Coronary Revascularization Study (NCT03491722) (891 patients) [16], the Jordan Atrial Fibrillation Study (NCT03917992) (2020 patients) [17], the Jordan COVID-19 Pandemic Acute Cardiovascular Events Study (NCT04368637) [19], the Statin Eligibility in AMI Patients registry (NCT03485742) (774 patients) [18], and the Study of Novel and Classical Risk Factors in Young Middle Eastern Women with ASCVD (NCT04975503) (627 participants) (Table 1) [15].

Data from the prospective cohort and the retrospective registries were combined into a single pooled analytical cohort. Uniform inclusion criteria and variable definitions were applied across all datasets, and analyses were conducted on the combined sample to ensure consistency and comparability.

Data collection was performed by trained research personnel utilizing standardized case report forms to promote methodological rigor and ensure data reliability. The collected variables encompassed demographic and anthropometric parameters, comprehensive medical histories, both traditional and emerging cardiovascular risk factors, classified into modifiable and non-modifiable domains, documented comorbid conditions, pharmacological regimens prescribed for secondary cardiovascular prevention, and patient survival outcomes assessed at one-year post index event.

### 2.2. Inclusion Criteria and Risk Factor Classification

The study cohort comprised patients with clinically confirmed ASCVD, operationally defined as a documented history of coronary artery disease (CAD), ischemic cerebrovascular events, peripheral arterial disease, or carotid artery atherosclerosis. The CAD subset included individuals diagnosed with chronic stable angina (CSA), acute coronary syndromes (ACS), encompassing both ST-segment elevation myocardial infarction (STEMI) and non-ST-segment elevation presentations, as well as cases identified via coronary computed tomography angiography (CCTA). Participants were stratified into three groups according to the number of SMuRFs: those without any SMuRFs, those with one to two SMuRFs, and those presenting with three to four SMuRFs.

Records with missing data in key study variables were excluded from the analysis. Only participants with complete information for the variables required for the present investigation were included in the final analytical cohort.

### 2.3. Definition of Standard Modifiable Risk Factors (SMuRFs)

SMuRFs were conceptualized as binary variables, defined in accordance with standardized diagnostic criteria and threshold values derived from established studies [11,20,21,22,23,24]. Dyslipidemia was determined based on either a prior clinical diagnosis, current use of lipid-lowering pharmacotherapy, or LDL-C ≥70 mg/dL (≥1.8 mmol/L) [25,26,27]. Type 2 diabetes mellitus (T2D) was classified through documented medical history, use of antidiabetic medications, or glycated hemoglobin (HbA1c) levels of 6.5% or higher. Hypertension (HTN) was identified via a known diagnosis, ongoing antihypertensive treatment, or the presence of newly elevated systolic (≥140 mmHg) and/or diastolic blood pressure (≥90 mmHg) recorded on multiple inpatient measurements. Current smoking status was assigned to individuals who reported regular tobacco use within the 12 months preceding study inclusion.

The study also incorporated the assessment of a conventional cardiovascular risk factor not encompassed within the SMuRF classification, specifically, a family history of premature cardiovascular disease. A positive family history was defined as the occurrence of a cardiovascular event in a first-degree male relative before age 55 or in a female relative before age 65. Additionally, MS was diagnosed in participants meeting at least three of the following criteria: the presence of hypertension, obesity, reduced high-density lipoprotein cholesterol (HDL-C) levels, defined as <40 mg/dL for men and <50 mg/dL for women, and elevated triglyceride concentrations exceeding 150 mg/dL. This definition was based on a modified NCEP ATP III framework tailored to the variables available across the pooled datasets. Hypertension was defined as a prior diagnosis, antihypertensive treatment, or repeated blood pressure ≥140/90 mmHg. Because waist circumference was not available in all registries, obesity was assessed using body mass index (BMI), with BMI ≥30 kg/m^2^ used as the obesity criterion in accordance with WHO classification. Fasting glucose and insulin resistance were not included because these variables were not consistently available across all data sources [28,29,30,31,32]. Smoking status was defined as current regular tobacco use, including cigarettes, waterpipe (shisha), or electronic cigarettes, on a daily or near-daily basis. Occasional or former use was not classified as regular tobacco use [33].

### 2.4. Ethical Considerations

The study was conducted entirely following the ethical principles outlined in the Declaration of Helsinki. Ethical approval was secured from the Institutional Review Board/Independent Ethics Committee of Istishari Hospital, Amman, Jordan. All participants provided written informed consent prior to enrollment. The study protocol has been registered with ClinicalTrials.gov (Identifier: NCT06199869).

### 2.5. Statistical Analysis

Descriptive and inferential statistical methods were employed for data analysis. Categorical variables are reported as frequencies and percentages, while continuous variables are presented as means ± standard deviations (M ± SD). The Chi-square test was used to assess the distribution of MS across groups stratified by the number of SMuRFs. Group comparisons of continuous variables were conducted using independent *t*-tests for normally distributed data and non-parametric tests for non-normally distributed data. Shapiro–Wilk was used to test the normality of the continuous variables. All tests showed *p*-values greater than 0.05, which indicated that they were normally distributed.

Univariate analysis was first used to explore associations between MS and demographic, clinical, and laboratory variables. Variables with significant associations were included in a multivariate logistic regression model to identify independent predictors of MS. Odds ratios (ORs) with 95% confidence intervals (CIs) were calculated, with statistical significance defined as *p* < 0.05. Medication use was analyzed by comparing individuals with and without MS using Chi-square tests for categorical variables and independent t-tests for continuous variables. All analyses were conducted using SPSS version 24, with a significance threshold of *p* < 0.05.

A formal sample size or power calculation was not performed. The study included all eligible patients available from the prospective cohort and retrospective registries who met the inclusion criteria and had complete data for the variables required for the present analysis. Therefore, the final sample size was determined by data availability.

## 3. Results

### 3.1. Metabolic Syndrome by SMuRFs Group

**Figure 1:**
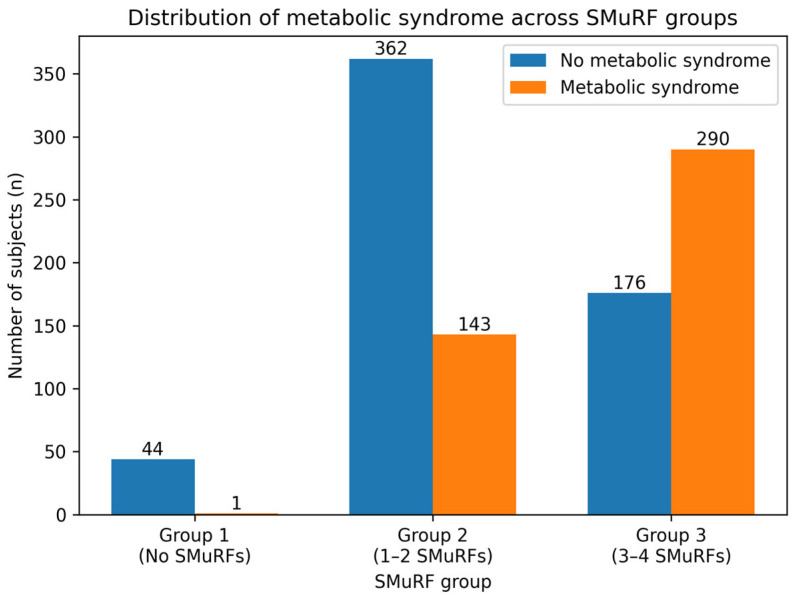
Sample Characteristics by MS Status.

As shown in Table 2, the prevalence of MS significantly increased with the number of SMuRFs (dyslipidemia, hypertension, diabetes mellitus, and smoking). As shown in Figure 1, MS prevalence increased progressively across SMuRF groups (2.2%, 28.3%, and 62.2% in Groups 1–3, respectively; *p* < 0.001).

The relatively small number of participants without SMuRFs (Group 1) may limit the statistical power and precision of comparisons involving this subgroup.

As shown in Table 3, patients with MS were older (60.5 ± 12.3) than those without MS (M = 54.6, SD = 12.3), *p* < 0.001. MS was more prevalent among males (63.4%) than females (36.6%), *p* < 0.001.

MS was significantly associated with a higher prevalence of hypertension (82.5%), diabetes (71.4%), dyslipidemia (87.1%), CKD (20.0%), and heart failure (37.3%) compared to those without MS (all *p* < 0.001). Interestingly, smoking was more common among patients without MS (45.4% vs. 36.9%, *p* < 0.05).

Metabolically, the MS group had higher BMI (31.0 vs. 28.1), triglycerides (182.7 vs. 111.8), and lower HDL (38.3 vs. 46.4), all *p* < 0.001. LDL-C, total cholesterol, and HDL were higher among those without MS (*p* < 0.05).

### 3.2. Medication Use by MS Status

As shown in Table 4, patients with MS were significantly more likely to use statins (91.7% vs. 85.1%, *p* < 0.005), low-dose acetylsalicylic acid (85.3% vs. 77.3%, *p* < 0.005), beta-blockers (83.3% vs. 69.2%, *p* < 0.001), and dual-antiplatelet therapy (50.5% vs. 44.3%, *p* < 0.05).

The most notable differences were in the use of oral hypoglycemic agents (53.2% vs. 20.6%) and insulin (26.0% vs. 8.1%), both significantly higher in the MS group (*p* < 0.001).

### 3.3. Predictors of MS in Patients with ASCVD

Logistic regression identified several independent predictors of MS, including advanced age, history of diabetes, hypertension, chronic kidney disease, heart failure, elevated body mass index (BMI), and high triglyceride levels (all *p* < 0.001) (Table 5).

Significant positive predictors of MS included a history of diabetes mellitus (*p* < 0.001), hypertension (*p* < 0.001), chronic kidney disease (*p* < 0.001), older age (*p* < 0.001), history of heart failure (*p* < 0.001), higher body mass index (BMI) (*p* < 0.001), and elevated triglyceride levels (*p* < 0.001). In contrast, higher HDL-C levels (*p* < 0.001) and smoking (*p* < 0.001) were negatively associated with MS.

## 4. Discussion

The results of the present study revealed a clear and statistically significant trend: the prevalence of MS increases systematically with the number of SMuRFs, namely dyslipidemia, hypertension, diabetes mellitus, and smoking. In particular, MS was detected in just 2.2% of patients with no SMuRFs but was present in 28.3% of those with one or two SMuRFs, ultimately rising to 62.2% in patients with three to four SMuRFs. This pattern highlights the harmonious and additive effects of cardiovascular disease comorbidities on metabolic status. The more SMuRFs, the more the likelihood of MS seems to increase, suggesting that instead of being isolated, independent disease burden determinants, such risk factors will tend to compound and worsen metabolic abnormalities.

These results agree with previous findings. A Brazilian study, for instance, highlighted that the clustering of conventional risk factors significantly increases the risk of cardiovascular events and metabolic disease [34]. In this line, Silveira Rossi, J.L., et al. (2021) [34] demonstrated that patients with a cardiovascular risk factor cluster are significantly more likely to develop MS, consistent with the increasing trend noted in our SMuRF cohorts. A plausible explanation for this finding is located in shared pathophysiological mechanisms of the elements of SMuRFs, i.e., chronic low-grade inflammation, insulin resistance, endothelial dysfunction, and excess sympathetic activity. Each risk factor on its own detracts from metabolic homeostasis. However, together, they are likely to accelerate the progression to full MS. Additionally, responses to one risk factor (e.g., oxidative stress caused by smoking) can enhance other metabolic disturbances, thus creating a self-reinforcing loop of metabolic damage [34,35]. The low incidence of MS in individuals who do not have SMuRFs, a mere 2.2%, highlights the strength of these risk factors as potent determinants of metabolic disorder. The observation provides confidence for anticipatory adjustment of risk factors, especially in individuals with one or two SMuRFs, to prevent the development of MS and related atherosclerotic disease.

This study identified several key discrepancies in clinical and metabolic profiles between patients with and without MS. In line with prediction, patients with MS were significantly older than their counterparts without MS, demonstrating age-related accumulation of risk determinants and physiological changes that enhance MS susceptibility. The mean age difference is based on findings of previous epidemiological studies, which consistently show increasing MS prevalence with advancing age due to the combined effect of modifiable risk factors and increasing metabolic dysregulation [36,37].

Interestingly, MS was more prevalent among men than women, a slight divergence from worldwide trends, indicating that women have a greater MS prevalence, particularly post-menopause, even among Middle Eastern women [38,39]. Differences in lifestyle, access to healthcare, and exposure to risk factors across regions may explain this disparity in our sample, possibly due to cultural or occupational factors that influence physical activity, smoking, and nutrition among men in the studied population.

In the current study, patients with MS had a significantly higher prevalence of hypertension, diabetes, and dyslipidemia, three of the constitutive elements of the syndrome. These observations add to the pivotal role of these illnesses in the pathophysiology of MS. They are consistent with the National Cholesterol Education Program Adult Treatment Panel III (NCEP ATP III) and the International Diabetes Federation (IDF) guidelines diagnostic criteria [40,41]. The relationship between chronic kidney disease (CKD) and heart failure is particularly evident, confirming past studies that have implicated MS in end-organ damage and poor cardiovascular events [42]. Both are probably the result of chronic vascular and metabolic strain from the components of MS, specifically hypertension and insulin resistance.

In this study, smoking was more prevalent in individuals without MS compared to those with MS. This negative relationship could at first appear unusual, as smoking is an established cardiovascular risk factor [43]. However, some research has observed that smokers have lower BMI, one of the basic diagnostic criteria for MS, which may partly account for this [44]. In addition, underreporting or misclassification of smoking status in clinical settings may affect observed patterns. Nevertheless, observation requires more investigation to clarify the interplay between smoking and MS clinical profiles in populations.

MS patients also had significantly higher BMI and triglyceride levels and lower HDL-C levels, all of which play a central role in MS diagnosis and were expected. LDL-C and total cholesterol levels were higher in the non-MS group. This apparently contradictory finding may be explained by a treatment effect. Participants with metabolic syndrome were more likely to receive statin therapy compared with those without metabolic syndrome, which could have contributed to lower LDL-C and total cholesterol levels in the MS group [45,46]. Previous research has also indicated that individuals with MS could have more frequent health contacts, which would result in improved lipid management despite increased cardiometabolic burden [46]. The family history of early cardiovascular disease was not significantly different across groups. This suggests that modifiable risk factors may influence the clinical differences observed more significantly than genetic factors alone. Thus, early lifestyle intervention is required regardless of family history.

The current study highlights that MS patients had a notably higher likelihood of being treated with major cardiovascular and metabolic medications than non-MS patients. Specifically, statin, acetylsalicylic acid, beta-blocker, and dual-antiplatelet therapy use was consistently more prevalent in the MS population, reflecting a more intense pharmacologic strategy directed at reducing their heightened cardiovascular risk. The findings are consistent with the evidence that shows a higher medication burden in MS patients because of the clustering of risk factors, including dyslipidemia, hypertension, and diabetes [47]. The higher rate of statin utilization in patients with MS is in line with guideline recommendations like those issued by the Adult Treatment Panel III (ATP III) and the International Diabetes Federation (IDF), which endorse the use of lipid-lowering treatment in individuals with metabolic dysregulation [40,41]. Similarly, the higher proportion of acetylsalicylic acid utilization is consistent with secondary prevention practices that are frequently applied to this cohort, given their elevated baseline risk of ASCVD [48].

The higher rate of beta-blocker usage may be reflective of the greater incidence of hypertension and heart failure in the MS cohort, both of which are known indications for beta-blockade therapy [49]. The higher rate of dual-antiplatelet therapy and P2Y12 inhibitors also supports the theory that MS patients are more likely to have underlying or prior cardiovascular events necessitating a comprehensive antithrombotic regimen [50].

The most significant differences were noted in the utilization of oral hypoglycemic agents and insulin, reflecting the close association of MS with glucose metabolism abnormalities, especially type 2 diabetes. This result is consistent with the commonly applied diagnostic criteria for MS, which include impaired glucose tolerance or diabetes, thus requiring heightened activity regarding glycemic control in this group [51,52].

The current research identified clinical and metabolic predictors that were independently associated with the prevalence of MS in individuals with ASCVD. Diabetes mellitus, hypertension, chronic kidney disease (CKD), older age, heart failure, elevated BMI, and elevated triglycerides were all positively correlated with MS, while elevated HDL-C and smoking were negatively associated with its occurrence. The most significant predictor of MS was a previous diagnosis of diabetes mellitus, consistent with the observed pathophysiological similarities between MS and type 2 diabetes. MS is primarily characterized by insulin resistance, and this strong correlation reinforces the existing literature highlighting diabetes as a primary component and consequence of metabolic dysfunction [53,54].

Similarly, hypertension and CKD were also predictive factors. This agrees with the fact that patients with MS have multiple interrelated comorbidities driven by endothelial dysfunction and chronic inflammation [55,56]. CKD’s role in this situation can be dual, as both a complication of MS-related conditions and a causative agent for the syndrome via dyslipidemia and defective glucose metabolism.

Age and heart failure were also positively associated with MS, possibly reflecting the cardiometabolic burden with advancing age and the role of MS in progressive cardiac dysfunction. Elevated BMI and triglycerides were anticipated results, as central obesity and hypertriglyceridemia are essential diagnostic criteria of MS [50]. These findings form the basis for emphasizing weight control and lipid reduction in MS prevention programs.

Notably, more elevated HDL-C levels were also inversely related to MS, corroborating its established protective effect on lipid metabolism and cardiovascular disease. Reduced HDL-C is a characteristic component of MS and has been repeatedly associated with heightened cardiometabolic risk [57]. The reported inverse association with smoking is counterintuitive and should be interpreted cautiously. While smoking is a well-established cardiovascular risk factor, its negative association with MS may be due to complex metabolic interactions or confounding factors not controlled in this model, e.g., lower BMI among certain smokers, or survival bias. Previous research has reported mixed findings on smoking and MS, with some observing neutral or even inverse associations in specific populations [43,58]. This requires further investigation within more diverse groups. In summary, these findings replace the multifactorial etiology of MS and identify several modifiable predictors that clinicians must closely monitor in individuals with ASCVD.

The results of this study also highlight several key differences between the studied Middle Eastern population and comparable Western populations. Although the worldwide trends for MS share some similarities, the prevalence, distribution of risk factors, and gender patterns of the present study are unique. Most significantly, MS was more prevalent in men in our cohort, contrary to the trend of most Western and even some regional studies, where postmenopausal women tend to have higher MS rates. This difference may be due to sociocultural and occupational factors specific to the Middle East region, such as gender differences in physical activity, smoking, diet, and use of preventive healthcare. In addition, despite the high rate of metabolic risk, the inverse association of smoking with MS may reflect regional differences in body composition, smoking intensity, or healthcare utilization. The overall clustering of SMuRFs and the early emergence of cardiometabolic burden in relatively young patients may also reflect regional lifestyle patterns, including the nutrition transition toward Western dietary patterns and low physical activity levels reported in the Middle East and North Africa region [59,60].

This study has several limitations that should be acknowledged. First, fasting blood glucose (glycemia) values were not available in the dataset. Given that metabolic syndrome is closely associated with insulin resistance and impaired glucose metabolism, the absence of glycemic data limited our ability to directly assess and compare glucose levels between groups. This may have restricted a more comprehensive evaluation of the metabolic profile of the study population. Second, the study’s observational nature limits conclusions about the relationships between metabolic syndrome and the associated clinical and metabolic variables. An important consideration is the overlap between SMuRFs and the diagnostic components of metabolic syndrome. Because hypertension, dyslipidemia, diabetes mellitus, BMI, triglycerides, and HDL-C were included in both the exposure and outcome definitions, the observed increase in MS prevalence with the number of SMuRFs was partly expected. Similarly, the identified predictors of metabolic syndrome should be interpreted with caution because they overlap with its defining criteria.

In addition to the lack of fasting glucose data and the definitional overlap between SMuRFs and metabolic syndrome components, this study has other limitations. Its observational design precludes causal inference. Residual confounding from unmeasured factors, such as diet, physical activity, and socioeconomic status, may have influenced the observed associations. Moreover, the use of BMI as a surrogate for central obesity, rather than waist circumference, may have resulted in the misclassification of metabolic syndrome, as BMI does not adequately capture visceral adiposity or fat distribution. Additionally, medication use, particularly statins and glucose-lowering agents, may have affected metabolic parameters. The exclusion of fasting glucose and diabetes status due to data unavailability may have further contributed to underestimation and potential misclassification of metabolic syndrome. Furthermore, including components of the metabolic syndrome definition as predictors in regression analyses may introduce circularity bias and inflate the observed associations. Therefore, these findings should be interpreted with caution. The lack of detailed stratified and adjusted analyses (e.g., by sex, age, or SMuRF burden) limits the depth of clinical interpretation and should be considered when interpreting the findings. The relatively small number of participants without SMuRFs may have reduced statistical power, potentially limiting the ability to detect significant differences and affecting the robustness of subgroup comparisons. Moreover, country-level identifiers were not uniformly available across all retrospective registries, which precluded a complete stratified analysis of metabolic syndrome prevalence by country. This limits the ability to fully explore regional heterogeneity within the pooled Middle Eastern cohort. As the study was based on registry data, the findings may not be fully generalizable to the broader population. In addition, the pooled dataset combined a prospective cohort with multiple retrospective registries, which may have introduced methodological heterogeneity in data collection, variable definitions, and follow-up procedures. This design also increases the risk of selection bias, as inclusion depended on available complete data, and information bias, due to differences in how variables were recorded across sources. Accordingly, the findings should be interpreted with caution.

These findings have several important implications. Firstly, they highlight the necessity for early screening and aggressive management of modifiable risk factors, particularly in individuals with one or two SMuRFs, to prevent progression to full MS. Secondly, gender-specific, culturally appropriate interventions must be developed, most significantly in light of this Middle Eastern sample’s greater percentage of MS men than women in Western samples. Additionally, clinicians need to monitor ASCVD patients for MS to guide pharmacologic therapy and implement lifestyle strategies aimed at weight loss, glycemic control, and lipid optimization. From a public health perspective, region-specific prevention initiatives targeting the unique sociodemographic and behavioral factors of Middle Eastern populations are necessary. Long-term outcomes and interventions targeting these individuals should be piloted in future research to prevent MS-related complications in ASCVD populations. Lastly, integrating MS screening into routine cardiovascular care pathways can improve outcomes and stem the region’s rising tide of cardiometabolic disease.

## 5. Conclusions

This study provides a comprehensive assessment of the burden, clinical profile, treatment patterns, and independent predictors of MS among Middle Eastern patients with established ASCVD. A clear and graded relationship was observed between the accumulation of SMuRFs and the likelihood of MS, underscoring the additive impact of hypertension, diabetes, dyslipidemia, and smoking on cardiometabolic health. Patients with MS tended to be older, predominantly male, and exhibited a higher prevalence of comorbid conditions, particularly chronic kidney disease and heart failure. Reflecting their greater cardiovascular risk, these patients were more likely to receive intensive pharmacological therapy. The identified predictors of MS highlight its complex, interconnected pathophysiology and reinforce the need for integrated, individualized risk assessment strategies in patients with ASCVD. Collectively, these findings emphasize the importance of early identification and comprehensive management of modifiable risk factors to mitigate the growing cardiometabolic burden in this high-risk population.

## Figures and Tables

**Table 1:** An Overview of Cardiovascular-Related Registries Involving Jordan and the Middle East.

Registry Name	Registry ID	Objective	Timeframe	Inclusion Criteria	Exclusion Criteria	Patient Characteristics	Cities/Centers Involved
Jordan SMuRF-less Study	NCT06199869	To evaluate cardiovascular risk factors in Jordanian adults beyond traditional SMuRFs.	10 January 2024–20 August 2024	Adults (≥18 years) diagnosed with ASCVD.	Congenital heart disease, pregnancy/lactation, or inability to provide consent.	Jordanian adults with ASCVD, focusing on non-traditional and emerging risk factors.	3 community hospitals and 6 tertiary centers across Jordan (MOH, university, and private sectors).
First Jordan PCI Registry	NCT01841346	To monitor outcomes, complications, and the effectiveness of PCI procedures in Jordan.	Ongoing	Adults (≥18 years) undergoing PCI for confirmed CAD.	Contraindications to PCI, refusal to participate, or serious interfering comorbidities.	Patients receiving elective or emergency PCI of various age groups.	Multiple hospitals across Jordan that perform PCI.
ASCVD Risk Factors in Young Women	NCT04975503	To identify both traditional and novel risk factors for ASCVD among young Middle Eastern women.	Long-term study	Females aged 18–45 years with or at risk for ASCVD.	Pregnancy, prior CABG or major cardiovascular events, and chronic systemic diseases.	Young Middle Eastern women at risk for cardiovascular disease.	Major urban medical centers in the Middle East (specific sites not listed).
Survival Post-Coronary Revascularization	NCT03491722	To examine long-term survival and outcomes after coronary revascularization procedures.	>10-year follow-up	Adults surviving ≥10 years after PCI or CABG.	Surgeries within the last 10 years or presence of terminal illness.	Long-term survivors of coronary artery disease in the Middle East.	Various Middle Eastern hospitals, including centers in Jordan.
Jordan Atrial Fibrillation Study	NCT03917992	To assess the prevalence, management strategies, and outcomes of atrial fibrillation in Jordan.	Ongoing	Adults (≥18 years) diagnosed with AF.	Non-cardiac arrhythmias, pregnancy, or contraindication to anticoagulation therapy.	AF patients, both new and previously diagnosed.	Jordanian hospitals with cardiology and arrhythmia units.
Statin Eligibility in AMI Patients	NCT03485742	To determine statin eligibility and treatment patterns among Middle Eastern AMI patients.	Not specified	Adults (≥18 years) with AMI who meet statin eligibility criteria.	Contraindication or history of adverse reaction to statins.	Middle Eastern adults hospitalized with AMI.	High-volume AMI centers across the Middle East.
Jordan COVID-19 Acute Cardiovascular Events Study	NCT04368637	To explore the impact of COVID-19 on acute cardiovascular events in Jordan.	During the COVID-19 pandemic	Adults (≥18 years) presenting with acute CV events during the pandemic.	Absence of CV events or confounding medical conditions.	Jordanian adults experiencing acute cardiovascular complications during COVID-19.	Hospitals across Jordan managing COVID-19 and cardiovascular emergencies.

Abbreviations: ASCVD: atherosclerotic cardiovascular disease; PCI: percutaneous coronary intervention; CAD: coronary artery disease; CABG: coronary artery bypass grafting; AF: Atrial fibrillation; AMI: acute myocardial infarction; CV: cardiovascular; COVID-19: coronavirus disease 2019; MOH: Ministry of Health.

**Table 2:** Metabolic syndrome of the total sample based on SMuRF groups (N = 1016).

Variable	Total Sample (n = 1016)	(G1): SMuRF-Less (n = 45)	(G2): One to Two SMuRFs (n = 505)	(G3): Three to Four SMuRFs (466)	*p* Value
Metabolic Syndrome					
No	582 (57.3)	44 (97.8)	362 (71.7)	176 (37.8)	<0.001
Yes	434 (42.7)	1 (2.2)	143 (28.3)	290 (62.2)	

SMuRFs: standard modifiable risk factors; G1: SMuRF-less; G2: 1–2 SMuRFs; G3: 3–4 SMuRFs. Values are presented as a number (%).

**Table 3:** Sample characteristics based on presence or absence of metabolic syndrome (MS) (N = 1059).

Variable	Total Sample (n = 1016)	(G1): Has MS (n = 434)	(G2): Do Not Have MS (n = 582)	*t* Test or *X*^2^, *p* Value
Age (years)	57.1 ± 11.5	60.5 ± 12.3	54.6 ± 12.3	7.6, *p* < 0.001
Sex				
Male	570 (56.1)	275 (63.4)	295 (50.7)	16.2, *p* < 0.001
Female	446 (43.9)	159 (36.6)	287 (49.3)	
History of hypertension	652 (64.2)	358 (82.5)	294 (50.5)	110.5, *p* < 0.001
History of diabetes mellitus	493 (48.5)	310 (71.4)	183 (31.4)	159.1, *p* < 0.0001
History of dyslipidemia	788 (77.6)	378 (87.1)	410 (70.4)	7.4, *p* < 0.005
Smoking	424 (41.7)	160 (36.9)	264 (45.4)	5.2, *p* < 0.05
Family history of premature CVD	421 (41.4)	173 (39.9)	248 (42.6)	*p* = 0.38
History of CKD	115 (11.3)	87 (20.0)	28 (4.8)	57.5, *p* < 0.001
History of Heart Failure	269 (26.5)	162 (37.3)	107 (18.4)	45.8, *p* < 0.001
Metabolic Syndrome BMI (kg/m^2^)	29.4 ± 5.4	31.0 ± 5.5	28.1 ± 5.0	8.8, *p* < 0.001
LDL-C mg/dL	102.8 ± 45.5	99.0 ± 44.4	106.1 ± 46.3	2.3, *p* < 0.05
Total cholesterol mg/dL	172.7 ± 53.5	169.3 ± 56.0	175.8 ± 51.0	1.7, *p* < 0.05
Triglycerides mg/dL	142.1 ± 119.4	182.7 ± 113.1	111.8 ± 97.8	9.8, *p* < 0.001
HDL-C mg/dL	42.6 ± 20.3	38.3 ± 10.9	46.4 ± 25.5	5.8, *p* < 0.001

G1: has MS; G2: no MS. Values are presented as number (%), or M ± SD. CKD: chronic kidney disease; CVD: cardiovascular disease; HDL: high-density lipoprotein; LDL-C: low-density lipoprotein.

**Table 4:** Medication Usage Among Participants With and Without MS (N = 1059).

Medication Used	Total Sample (n = 1016)	(G1): Has MS (n = 434)	(G2): Do Not Have MS (n = 582)	*X*^2^, *p* Value
Statins	893 (87.9)	398 (91.7)	495 (85.1)	10.3, *p* < 0.005
low-dose acetylsalicylic acid	820 (80.7)	370 (85.3)	450 (77.3)	10.1, *p* < 0.005
clopidogrel bisulfate	498 (49.0)	205 (47.2)	293 (50.3)	*p* = 0.18
P2Y12 inhibitors	72 (7.1)	45 (10.4)	27 (4.6)	12.4, *p* < 0.001
Dual-antiplatelet therapy	477 (46.9)	219 (50.5)	258 (44.3)	3.8, *p* < 0.05
Beta blockers	760 (74.8)	357 (83.3)	403 (69.2)	22.3, *p* < 0.001
Oral hypoglycemic agents	351 (34.5)	231 (53.2)	120 (20.6)	116.9, *p* < 0.001
Insulin	160 (15.7)	113 (26.0)	47 (8.1)	116.9, *p* < 0.001

Values are presented as a number (%); MS: Metabolic Syndrome; G1: MS; G2: no MS.

**Table 5:** Predictors of metabolic syndrome based on the Logistic Regression model.

Predictor	Wald	Odds Ratio	95% CI	*p* Value
Age	5.3	1.02	1.003–1.037	0.021
History of Hypertension	32.1	3.07	2.08–4.5	0.0001
Smoking	5.3	0.62	0.41–0.93	0.021
History of Diabetes Mellitus	53.7	4.01	2.77–5.82	0.0001
CKD	8.1	2.43	1.32–4.47	0.004
History of Heart Failure	6.8	1.71	1.41–2.55	0.009
BMI	24.9	1.1	1.05–1.13	0.0001
Triglycerides	12.7	1.004	1.002–1.006	0.0001
HDL-C	16.9	0.97	0.95–0.98	0.0001

CKD: Chronic Kidney Disease; BMI: Body Mass Index; HDL-C: high-density lipoprotein; CI: Confidence Interval.

## Data Availability

The datasets generated and analyzed during the current study are not publicly available but may be obtained from the corresponding author upon reasonable request.
